# Aerobic Microbiological Spectrum and Antibiotic Resistance in Children Operated for Anorectal Abscesses

**DOI:** 10.3390/jcm13082414

**Published:** 2024-04-20

**Authors:** Dzhevdet Chakarov, Elena Hadzhieva, Yordan Kalchev, Dimitar Hadzhiev

**Affiliations:** 1 Section of General Surgery, Department of Propedeutics of Surgical Diseases, Faculty of Medicine, Medical University of Plovdiv, 4001 Plovdiv, Bulgaria; dzhevdet.chakarov@mu-plovdiv.bg (D.C.); dimitar.hadzhiev@mu-plovdiv.bg (D.H.); 2 First Clinic of Surgery, University Hospital St. George, 4001 Plovdiv, Bulgaria; 3 Department of Medical Microbiology and Immunology “Prof. Dr. Elissay Yanev”, Faculty of Medicine, Medical University of Plovdiv, 4002 Plovdiv, Bulgaria; yordan.kalchev@mu-plovdiv.bg; 4 Laboratory of Microbiology, University Hospital St. George, 4002 Plovdiv, Bulgaria

**Keywords:** anorectal abscess (ARA), microbial spectrum, aerobes, anorectal fistula

## Abstract

**(1) Background**: Anorectal abscesses are a relatively rare pathology in childhood. Most often, male children under 1 year of age are affected. The importance of microbiological examination for the diagnosis and treatment of such patients remains debatable among surgeons, resulting in scarce data being available in the literature. We aimed to identify the aerobic microbiological spectrum and antibiotic resistance of isolates in children undergoing operation to treat anorectal abscesses. **(2) Methods**: We performed a case series of 102 children diagnosed and operated for anorectal abscesses over a period of 10 years (2010–2019). Purulent wound exudate was used for microbiological evaluation, which was subsequently cultured on 5% sheep-blood agar and eosin–methylene blue agar. For microbiological identification, conventional biochemical tests and semi-automated (API 20, bioMerieux, Marcy-l’Étoile, France) tests were used, as well as automated systems (Vitek-2 Compact, bioMerieux, France). Antimicrobial susceptibility testing was performed by the disk diffusion method of Bauer–Kirby and by determining the minimal inhibitory concentrations for glycopeptides. The results were interpreted according to the EUCAST standard for the corresponding year. **(3) Results**: Microbiological testing in children operated for anorectal abscesses mainly identified the gut commensals that normally reside in the rectal mucosa. Monocultures were found in just over half of the cases. *Escherichia coli*, *Klebsiella pneumoniae complex*, and *Proteus mirabilis* were the most frequently isolated. In addition, *Staphylococcus aureus* was found in 7% of patients. In Gram-negative bacteria, antibiotic resistance was most often observed in penicillins, cephalosporins, sulfonamides, and fluoroquinolones. **(4) Conclusions**: The increasing rates of antimicrobial resistance impose the need for the local monitoring of circulating commensal bacteria associated with anorectal abscesses in children to guide antibiotic therapy when indicated.

## 1. Introduction

Anorectal abscesses (ARAs) are among the most common inflammatory diseases in the anorectal area in adult patients and, although less common, the disease is also present in childhood [1]. The lower incidence of the disease in children could be attributed to the anatomical, physiological, and behavioral differences between children and adults [2,3,4]. The disease affects predominantly male children under 1 year of age but can be found in both sexes up to 18 years of age, possibly in association with Crohn’s disease, Hirschsprung’s disease, penetrating trauma, and immunosuppression [5,6,7].

ARAs are characterized by the accumulation of pus in the anorectal region. The disease in healthy children is usually self-limiting and often requires only surgical treatment. However, ARAs pose the risk of serious complications, including fistula formation and systemic infection, especially when risk factors are present, thus making the therapeutic approach more challenging [8,9]. Complications and the development of perianal fistula can reach 24% when associated with colonic pathology. The main trigger for the onset of ARA, in both pediatric and adult patients, is the infection of the pararectal tissues. While the cryptoglandular theory explains the infection in more than 90% of adults, it has inconsistencies and points of controversy, especially in younger children [10,11,12].

The anorectal region has a unique microbial commensal microbiota, composed of both aerobic and anaerobic bacteria that contribute to the pathogenesis of ARAs’ formation. Studies on the bacteria associated with ARA have provided some insights into the microbial landscape of the anorectal area, demonstrating a diverse community of bacteria that could contribute to the development of these abscesses. Among these, organisms such as *Escherichia coli*, *Staphylococcus aureus*, *Streptococcus* spp., *Bacteroides* spp., and *Fusobacterium* spp. are frequently implicated. ARAs are often reported to be polymicrobial in nature, involving complex interactions between multiple microbial species. This polymicrobial aspect complicates the diagnosis and treatment, as different bacteria may have varying susceptibility to antibiotics [13,14,15,16,17,18,19].

However, in the medical literature, studies of the microbiological spectrum of aerobic agents of ARA in children are scarce. This demonstrates that ARAs in children still lack clarity in terms of their etiology and diagnosis, and the therapeutic approaches. Understanding the microbial causes of anorectal abscesses is crucial for developing effective treatment strategies and improving patient outcomes. The etiology and antimicrobial resistance of isolates can enhance the effective institutional antibiotic policy and the selection of empiric treatment. The following case series aims to determine the aerobic microbiological spectrum and antibiotic resistance of isolates in children operated for anorectal abscesses. 

## 2. Material and Methods

### 2.1. Selection of Patients

We performed a case series involving 102 children operated for ARA for a period of 10 years (2010–2019). The study was designed to collect data by reviewing medical records that involved sex, age, microbiological results, and antimicrobial susceptibility reports. All children were hospitalized and treated in the First Surgery Clinic and the Paediatric Surgery Clinic of “St. George” University Hospital. We defined the study group based on the following inclusion and exclusion criteria:

Inclusion criteria:

- All children with ARA aged from 0 to 18 years.

Exclusion criteria:

- Patients with anorectal fistula (ARF);
- Cases with suppurated cusp of pilaris regio sacralis;
- Patients with anorectal form of Crohn’s disease;
- Cases with Proctitis ulcerohaemorrhagica chronica;
- Cases with chronic specific diseases (TBC, actinomycosis, etc.), leading to the appearance of ARA;
- Patients with Bartolinitis acuta;
- Cases with Pyodermia fistulosa;
- Cases with pararectal purulent inflammation of non-cryptoglandular origin (perianal skin furuncle, suppurative perianal atheroma, purulent hidradenitis, suppurative teratoma).

### 2.2. Microbiological Examination

For the microbiological examination, purulent wound exudate was taken with a dry sterile swab in a transport medium (Amies, Biolife Italiana S.r.l., Milan, Italy), during the operative intervention, at the initial incision. Inoculation was carried out using 5% sheep-blood agar (Diachim, Sofia, Bulgaria) and a selective medium for Gram-negative bacteria–eosin-methylene blue agar (Diachim, Sofia, Bulgaria). The agars were incubated for 18–22 h at 36–37 °C. Conventional manual biochemical tests, semi-automated (API 20, bioMerieux, Marcy-l’Étoile, France), and automated systems (Vitek-2 Compact, bioMerieux, Marcy-l’Étoile, France) were used for microbiological identification. All cultured microorganisms were subjected to identification and testing to determine antimicrobial susceptibility to antibiotic agents.

Antimicrobial susceptibility was determined via the Bauer–Kirby disk diffusion method, and an automated system was used to establish minimum inhibitory concentrations (Vitek-2 Compact, bioMerieux, Marcy-l’Étoile, France). When interpreting the results of the antimicrobial susceptibility tests, the current version of the EUCAST standard for the relevant year was used.

We did not perform other follow-up microbiological tests because we did not aim to dynamically follow the changes occurring in the aerobic bacterial microbiota. We also aimed to investigate the aerobic microbiological landscape in the operated children with ARA; therefore, no cultures were created to detect anaerobic bacteria.

The following study endpoints encapsulate our primary objectives:

*Demographic Characteristics*

- Analysis of age profile and gender distribution among children with ARA.

*Microbiological Profile of ARA Cases*

- Investigation of the structure of microbial isolates as monoculture and microbial associations.
- Distribution of the etiological structure of ARA in children.
- Data on antimicrobial resistance in the most common isolates.

### 2.3. Statistical Methods

The statistical package SPSS Statistics v.26 (IBM Corp., Chicago, IL, USA) was used for the statistical processing of the results. Numerical values are stated as mean ± standard deviation and categorical data are presented as frequency (n) and percentage (%). 

## 3. Results

**Figure 1:**
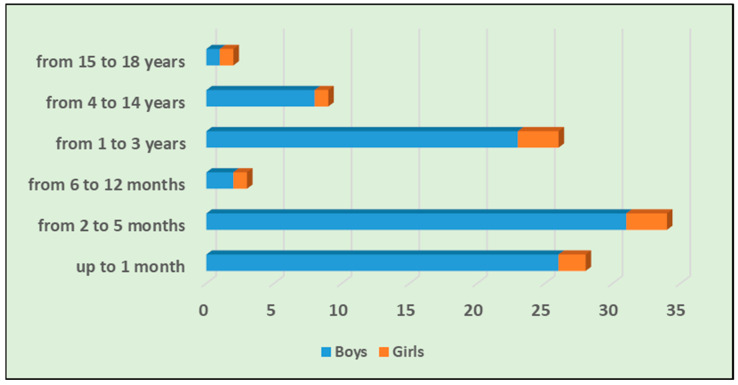
Distribution of the age and sex of operated children with ARA.

Among the children with ARA, 91 (89.2%) were boys and 11 (10.8%) were girls, as shown in Table 1. The mean age of the children included in the study was 1.6 years (SD ± 3.15). The mean age of boys (*n* = 91) was 1.45 years, SD ± 2.93, while the mean age of girls (*n* = 11) was 2.79 yrs, SD ± 4.43. Of the children that were operated on, the youngest patient was 1 month old and the oldest was 17 years old. The most affected age group among children was neonates and infants, with a total of 65 patients (63.7%), as shown in Figure 1. In infancy, seven children (6.9%) were found to have concomitant diseases, most of them with a congenital genesis.

The distribution of operated children with ARA according to age and sex is presented in Figure 1.

The results obtained from the bacteriological cultures were divided into three main groups: monocultures, mixed cultures, and sterile cultures (Table 2):

Table 3 presents the structure of microbial isolates as a monoculture. The most commonly isolated organism in the monoculture was *E. coli* (56%), followed by *Klebsiella* spp. (20%). *S. aureus* was cultured in 12.5% of patients.

The resulting microbial associations are presented in Table 4. The most frequently observed microbial association was between *E. coli* and *P. mirabilis* and between *E. coli* and *K. pneumoniae complex*. In addition, *E. coli* participated most often in microbial associations.

The microorganisms that were most represented, either as a monoculture or in microbial associations, were *E. coli*, *K. pneumoniae complex*, *P. mirabilis*, and *S. aureus*, as shown in Table 5.

Table 6 presents the data on the determination of the antimicrobial sensitivity of the four most frequently presented microorganisms in the etiological structure of ARA in children during the observation period. The highest levels of resistance in *E. coli* isolates were found for ampicillin, amoxicillin/clavulanic acid, and piperacillin. Resistance levels to third-generation cephalosporins reached 28%. Resistance to trimethoprim/sulfamethoxazole was found in 27% of the isolates. There was 100% sensitivity to carbapenems.

For *K. pneumoniae*, resistance to amoxicillin/clavulanic acid was recorded in 36% and resistance to piperacillin was recorded in 27%. Resistance to third-generation cephalosporins reached 22%. Of the isolates, 9% were resistant to levofloxacin. Sensitivity to carbapenems, piperacillin/tazobactam, cefoperazone/sulbactam, amikacin, and trimethoprim/sulfamethoxazole was maintained.

In *P. mirabilis*, resistance to ampicillin and amoxicillin/clavulanic acid was demonstrated in 29% and resistance to piperacillin and cefoperazone/sulbactam was demonstrated in 25%. ESBL producers were detected in 14%. Sensitivity to carbapenems, piperacillin/tazobactam, and cefoperazone/sulbactam was maintained. Susceptibility to carbapenems, the fluoroquinolones levofloxacin and ciprofloxacin, as well as to amikacin and trimethoprim/sulfamethoxazole, was observed.

Penicillin resistance levels in *S. aureus* reached 88%. MRSA isolates were found in 29%. Tested microorganisms showed sensitivity to trimethoprim/sulfamethoxazole, clindamycin, glycopeptides, linezolid, and tigecycline.

## 4. Discussion

The lack of clarity and consensus on the etiopathogenesis and the means of occurrence of ARAs in childhood generates controversial questions about their genesis and therapeutic management. Whenever a clinical analysis of the aerobic microbiological landscape in children with ARA is performed, one must consider the age-specific anatomical differences and age-related changes in the gut microbiome. It is well established that weak immunity in early childhood leads to decreased local protective factors, which is an important prerequisite for the development of a purulent inflammatory process [20,21]. This explains the poor resistance of perianal tissues in childhood to purulent infection. The cryptoglandular origin of ARA explains the penetration of the infection in more than 90% of adult patients very well, but regarding the same theory, there are several points of discussion and inconsistencies in children. According to this theory, the origin of purulent inflammation is from the anal glands, but these are functionally inactive in children and only begin to secrete after puberty. Some authors believe that ARA infections in childhood often result from penetrating rectal wall injuries, local pyoderma, skin injuries, cryptitis, proctitis, rhagades, congenital anorectal malformations, anomalies of the ducts of the anal glands, transient intestinal disorders, etc. [22]. The vulnerability of the epidermis, especially in infancy, with the presence of still-underdeveloped basement membranes, allows for lesions, even from microtrauma, to provide a gateway opportunity for infection.

It should be emphasized that electronic databases of medical publications on the aerobic microbial spectrum in operated children with ARA are very scarce. Logically, a bacteriological examination of operated children with ARA will mainly detect the microbial agents that are normally present in the rectal lumen. In a few clinical cases, microbiological analysis may reveal a mixed infection consisting of microbial associations. The present study found evidence of monocultures to be more frequent among aerobic opportunistic enteric bacteria. According to Abercrombie JF et al., the clinical and microbiological characteristics of pediatric perianal abscesses are like those of adults [23]. According to our data, the most common microorganism with an undisputed leading role as the causative agent of ARA in childhood is *E. coli*, with a relative contribution of about 40%. Other common representatives of the aerobic microbiological landscape are *K. pneumoniae complex* (9.6%) and *P. mirabilis*—7.0%. Of interest is the study by Zhu Y et al., reporting that *K. pneumoniae* is the most common pathogen in perianal abscess in infants aged less than 3 months and is usually resistant to ampicillin and nitrofurantoin. Since perianal abscess in infants younger than 3 months is a disease with a tendency to self-heal, a simple surgical intervention with the synchronous administration of antibiotics is suggested as the optimal treatment [24]. For example, A. Niyogi et al. [3] found the presence of enteric microorganisms in a large proportion of recurrent ARA patients. According to Tan Tanny SP. et al., the presence of enteric microorganisms in microscopic and culture studies in pediatric perianal abscess was not associated with fistula formation nor with abscess recurrence. Their studies also showed a predominance of enteric microorganisms in these patients [25].

Our results show that *S. aureus* (7.0%) is not an uncommon isolate of ARA in childhood, and an intriguing observation is that it occurred only as a monoculture and never in microbiological association. It is important to keep in mind that, in children, it is not uncommon to mix true ARA (infection from the intestinal lumen) with other, similar purulent inflammations that are nearby, such as furuncles, abscesses, and purulent adenitis. (infection from the skin and its appendages) [26,27]. These latter non-cryptoglandular purulent-inflammatory processes are not infrequently assumed to be ARAs, and are significantly induced by microorganisms of the genus Staphylococcus or with the major involvement of *S. aureus*. This suggests the possibility of inaccurate diagnosis in childhood with the presence of infected furuncles, skin abscesses, purulent adenitis, local pyodermas, etc., with *S. aureus* as the main causative agent. These conditions may be mistakenly categorized as ARA.

Questions about the need to establish an etiologic spectrum and antimicrobial resistance in ARA are controversial. There are conflicting statements about the clinical effect of identifying the specific microbiological causative agent and its antibiotic treatment, as some physicians refrain from using antibiotics in patients with ARA. It should be noted that all authoritative surgical opinions of the coloproctology community currently have a reserved and cautious attitude regarding the use of antibacterial therapy in ARA, as this is only recommended in a septic and complicated clinical course [28]. According to the American Society of Coloproctologists (ASCRS) medical standard, antibacterial treatment in ARA is selectively required only in the setting of complications with diffuse pararectal cellulitis, the presence of a systemic septic reaction, and evidence of immunosuppression in patients. According to them, the use of antibiotics for uncomplicated ARA in healthy children has no application, does not improve the healing process, and does not reduce new recurrences [29]. The SICCR and the German S3 guideline have similar views, generally considering antibiotics to have a limited role in trivial ARA, believing they should not be used in uncomplicated ARA, and considering them to be inappropriate [30,31,32,33]. For example, Shaughnessy MP et al. concluded that the results of microbiological cultures are of limited utility in the treatment of pediatric pilonidal, ischial, and perianal abscesses, as they do not appear to alter treatment, and omitting culture collection is not associated with failure of surgical treatment [34]. M.S. Brar et al. [35] consider that, in the postoperative period, antibacterial therapy is important to prevent fistula formation, and they recommend it for a 7–10-day course. However, given the short-term follow-up of the study, it is unclear how routine antibiotic administration will reduce ARF formation in the long term. V. Mocanu et al. [36], in a 2019 study, reported that antibiotic therapy after the incision and drainage of the ARA was associated with 36% lower odds of fistula formation. The authors considered that an empirical 5–10-day course of antibiotics after surgical drainage may avoid subsequent fistula formation in healthy children, although the quality of the evidence was low. Further randomized trials are needed to fully clarify the role, duration, and type of antibiotics best suited for postoperative ARF prophylaxis after drainage in perirectal abscesses. According to U. Sozener et al. [37], antibiotic treatment after anorectal abscess drainage has no protective effect on the risk of fistula formation.

However, it should be noted that the supporters of such controversial statements are significantly fewer than those supporting the opposite view.

Analyzing the data from the 10-year microbial monitoring in operated children with ARA, and conditionally dividing it into two periods of 5 years, the following conclusions can be drawn:

- A periodic increase in the frequency of enteric bacteria was found.
- During the first 5-year period (2010–2014), there was an increase in the frequency of staphylococci, particularly Staphylococcus aureus, with a subsequent large decrease during the last period.
- In the second 5-year period (2015–2019), an increase in the number of microbial associations was observed, which indicates an increase in the diversity of microorganisms found.
- The various data regarding the presence of sterile cultures are interesting, with 34% being found in the first period and 14.1% in the last period.

The analysis of the obtained results revealed the most pronounced resistance to the group of penicillins, cephalosporins, sulphonamides, and fluoroquinolones. Most likely, this resistance is a consequence of the excessive administration of certain antibiotic agents and the mechanisms of transfer between bacteria of genes mediating antimicrobial resistance, allowing for rapid dissemination.

We believe that clarifying the microbial sensitivity is important in the application of appropriate empirical therapy when necessary and to initiate optimal antimicrobial treatment in a timely manner after the antibiogram result is established. Therefore, the availability of specific antimicrobial sensitivity data of isolates in operated children with ARA can help to make timely and accurate choices for starting empirical antibacterial therapy and, consequently, avoid the administration of drugs to which the microorganisms are resistant [34,37]. Data on the persistence or increase of resistance levels require optimization, or a re-evaluation of the currently performed antibacterial treatment is required, to build a newer and more advanced antibiotic therapy concept for operated children with ARA. Recommendations to improve the efficacy of antimicrobial therapy and a new approach to monitor and limit resistance manifestations are also needed.

## 5. Conclusions

The *E. coli* and *K. pneumoniae* complex that are commensals in the gut were most frequently found in children with ARA. A common isolate was *S. aureus*, which plays a controversial role in the occurrence of ARA. The highest levels of resistance in *Enterobacterales* were found to occur against penicillins and cephalosporins. Increasing levels of antimicrobial resistance necessitate monitoring of the etiological spectrum and antimicrobial resistance of isolates in ARA in children to guide the choice of antibiotic therapy when indicated. We suggest that monitoring the etiological spectrum and antimicrobial resistance are important factors for improving the care of children with this rare pathology. Furthermore, we believe that, with this research, we could enrich the literature with important epidemiological data on the current etiological spectrum and antimicrobial resistance of bacterial cultures in children with ARA. 

## Figures and Tables

**Table 1:** Characteristics of the studied contingent.

	N	Mean Age	Standard Deviation
Boys	91	1.45	2.93
Girls	11	2.79	4.43

**Table 2:** Distribution of microbiological results by groups.

	N	%
Monocultures	64	62.7
Mixed cultures	12	11.8
Sterile	26	25.5
Total	102	100

**Table 3:** Data on the etiological structure of monocultures.

Structure of Established Agents as a Monoculture	Frequency	Percent
*E. coli*	36	56.3%
*Klebsiella* spp. (*K. pneumoniae complex*, *K. oxytoca*, *K. aerogenes*)	13	20.2%
*S. aureus*	8	12.5%
*Enterococcus faecalis*	2	3.1%
*Enterobacter cloacae complex*	1	1.6%
*Proteus mirabilis*	1	1.6%
*Citrobacter freundii*	1	1.6%
*Serratia marcescens*	1	1.6%
*Streptococcus beta-haem. Group A*	1	1.6%
Total	64	100%

**Table 4:** Data on the etiological structure of microbial associations.

Microbial Association	Frequency	Percent
*E. coli* + *P. mirabilis*	4	33.3%
*E. coli* + *K. pneumoniae complex*	2	16.7%
*E. coli* + *E. faecalis*	1	8.3%
*E. coli* + *E. coli ESBLs* (+)	1	8.3%
*Proteus* spp. + *K. aerogenes*	1	8.3%
*P. mirabilis* + *E. cloacae complex*	1	8.3%
*Pseudomonas aeruginosa* + *P. mirabilis*	1	8.3%
*E. cloacae complex* + *P. aeruginosa*	1	8.3%
Total	12	100%

**Table 5:** Data on the etiological structure of ARA in children.

Structure of Isolates in Children	Frequency	Percent
*E. coli*	45	39.5%
*K. pneumoniae complex*	11	9.6%
*P. mirabilis*	8	7.0%
*S. aureus*	8	7.0%
*E. cloacae complex*	3	2.6%
*E. faecalis*	3	2.6%
*K. oxytoca*	3	2.6%
*K. aerogenes*	2	1.8%
*P. aeruginosa*	2	1.8%
*Streptococcus beta-haem. Group A*	1	0.9%
*S. marcescens*	1	0.9%
*C. freundii*	1	0.9%
Sterile cultures	26	22.8%
Total	114	100%

**Table 6:** Antimicrobial resistance (%) in the most common ARA isolates in children.

	*E. coli*	*K. pneumoniae* *Complex*	*P. mirabilis*		*S. aureus*
AMP	60	i.r.	29	PEN	88
PIP	51	27	25	FOX	29
TZP	19	0	0	ERY	17
AMC	58	36	29	CLI	0
SCF	6	0	25	AMK	14
FOX	14	9	14	SXT	0
CTX	28	20	14	VAN	0
CRO	28	20	14	TEC	0
FEP	21	20	14	LZD	0
IPM	0	0	0	TGC	0
MEM	0	0	0		
AMK	5	0	0		
CIP	9	9	0		
LVX	11	0	0		
SXT	27	0	0		
TGC	7	-	-		

AMP—ampicillin; PIP—piperacillin; TZP—piperacillin/tazobactam; AMC—amoxicillin/clavulanic acid; CSF—cefoperazone/sulbactam; FOX—cefoxitin; CTX—cefotaxime; CRO—ceftriaxone; FEP—Cefepime; IPM—imipenem; MEM—meropenem; AMK—Amikacin; CIP—ciprofloxacin; LVX—levofloxacin; SXT—trimethoprim/sulfamethoxazole; TGC—tigecycline; PEN—penicillin; ERY—erythromycin; CLI—clindamycin; VAN—vancomycin; TEC—teicoplanin; LZD—linezolid; i.r.—intrinsic resistance.

## Data Availability

Data are available from the corresponding author on a reasonable request.
